# Does Infertility Stress Impair Sexual Function in Infertile Women and Men? A Cross-Sectional Study in Iran

**DOI:** 10.3389/fmed.2022.896538

**Published:** 2022-06-22

**Authors:** Samira Amraei, Parvin Abedi, Roshan Nikbakht, Mitra Tadayon, Elham Maraghi

**Affiliations:** ^1^Reproductive Health Promotion Research Center, Ahvaz Jundishapur University of Medical Sciences, Ahvaz, Iran; ^2^Midwifery, Ahvaz Jundishapur University of Medical Sciences, Ahvaz, Iran; ^3^Gynecology, Ahvaz Jundishapur University of Medical Sciences, Ahvaz, Iran; ^4^Biostatistics, Ahvaz Jundishapur University of Medical Sciences, Ahvaz, Iran

**Keywords:** infertility stress, female sexual function, male sexual function, infertile men, infertile women

## Abstract

**Background:**

The issue of infertility is a worldwide challenge, estimated to affect around 50 million couples. This study aimed to evaluate the relationship between infertility stress and sexual function in men and women with infertility.

**Methods:**

In this cross-sectional study, 300 men and women with infertility were recruited. A demographic questionnaire, the Fertility Problem Inventory, the Female Sexual Function Scale (FSFI), and the International Index of Erectile Function (IIEF) were used to collect data. Independent *t*-test, Chi-square, and linear and logistic regressions were used to assess the data.

**Results:**

The total score of sexual function in women and men was 22.18 ± 7.86 and 57.7 ± 17.8, respectively. Infertility duration and the ability to pay for the costs of infertility treatment had a significant relationship with sexual function in women. A significant association was found between communication concerns and sexual function scores in men with infertility. Infertile women had more sexual concerns, while infertile men had more communication concerns.

**Conclusion:**

This study showed that women with infertility had more infertility stress than men with infertility. Women with higher infertility duration and the inability to pay for the infertility treatment costs impaired sexual function. Women with infertility had more sexual concerns, while Men with infertility had more communication concerns. Policymakers need to consider strategies such as counseling for men and women with infertility to help them cope with their problems, especially their psychological problems. The lack of random enrollment of participants and lack of control group were the limitations of this study.

## Background

Infertility is a challenging worldwide issue which is estimated to affect around 50 million couples (1). In other words, every sixth couple is affected by infertility (2). The prevalence of primary clinical infertility in Iran was estimated to be 20.2% in 2019 (3). The causes of infertility can be listed as follows: non-ovulation or irregular ovulation (20%), tubal factor (30%), semen migration factor (10%), and male factor (30%) (2).

Infertile couples may experience psychological problems such as stress and depression. A study in Sweden showed that 10.9% of infertile women and 5.1% of men suffered from depression while 14.8% of women and 4.9% of men suffered from anxiety (4). A study by Patel et al. (5) on 300 infertile women showed that the prevalence of stress was 80%, and there was a significant association between stress and infertility type.

Infertility stress may impair sexual function and satisfaction in men and women with infertility. A study by Facchin et al. (6) showed that infertile women with higher infertility stress were more likely to have sexual dysfunction. Also, there is evidence that shows that the male partners of infertile women experience more stress and higher sexual dysfunction (7). Sexual function in infertile women may depend on the fertility of their partners. A study by Ghavi et al. (10) showed that sexual assertiveness in women with a fertile partner was higher in comparison with women having an infertile partner, and sexual burnout in women with an infertile partner was higher (8). Furthermore, infertile women experience more stress and depression and have a lower sexual quality of life (9), and the more years of infertility and its treatment, the lower the sexual function in women (10). Winkelman et al. (11) in their study showed that when the cause of infertility is the female factor, and in women younger than 40 years with primary infertility, the sexual function is more impaired.

A study in Iran showed that the prevalence of sexual dysfunction among infertile women was 64.3%, with sexual desire being more impaired than other sexual domains (12). Despite these studies, there is scarce information about the relationship between infertility stress and sexual function in infertile men and women.

### Objectives

This study was designed to evaluate the relationship between infertility stress and sexual function in men and women with infertility using validated questionnaires.

## Materials and Methods

This was a cross-sectional study in which 150 infertile women and 150 infertile men were recruited from the Infertility Center in Imam Khomeini Hospital in Ahvaz, Iran. Women and men with the following criteria were enrolled in this study: literate, married and having sexual intercourse at least for one year, and being diagnosed with primary infertility diagnosed by a gynecologist. Women and men with known physical or psychological disorders were excluded from the study.

### Ethical Consideration

The protocol of this study was approved by the Ethics Committee of Ahvaz Jundishapur University of Medical Sciences (Ref. ID: IR.AJUMS.REC.1398.144). All participants provided written informed consent prior to data collection. All participants were assured about the confidentiality of data. For preserving the privacy of participants, data were collected in a private room in the hospital and only one of the researchers (SA) was available in case participants had any questions.

### Sampling

The following formula was used for computing the sample size:


(1) $$\begin{array}{r} {\left(\left(Z_{1-\frac{\alpha }{2}}+Z_{1-\beta }\right)/C\right)}^{2}+3,C=0.5^{*}ln\left[\left(1+r\right)/\left(1-r\right)\right] \end{array}$$

In this formula, the power of the study was set at 90%, α = 0.05, *r* = 0.28 (minimum correlation coefficient between infertility stress and sexual function in infertile men and women from a pilot study). The total sample of women and men was set to be 130, and assuming 15% for non-responders, the total number of samples was estimated to be 300 (150 for women and 150 for men). The convenient sampling method was used for sampling.

### Measurements

In this study, we used four questionnaires to collect data. A demographic questionnaire which included questions about the participant's age, age of the spouse, duration of infertility, educational attainment, employment status, employment status of the spouse, economic status, and the ability to pay for the infertility treatment costs was designed by the researchers. The validity of the demographic questionnaire was approved using content validity. This questionnaire was checked by six faculty members and gynecologists.

The Fertility Problem Inventory was used for measuring the infertility stress of infertile women and men. This scale was developed by Newton et al. (13) and has 46 questions in five categories including social concerns, sexual concerns, communication concerns, child-free lifestyle, and need for parenthood (13). The answers to questions are rated from strongly disagree to strongly agree, and a six-item Likert scale is used for scoring the items, with one indicating “strongly disagree” and six indicating “strongly agree”. The minimum and maximum scores of this questionnaire are 46 and 276, respectively. The scores between 46 and 92 indicated low infertility stress, 92–184 show moderate infertility stress, and scores>184 represent high infertility stress. This questionnaire was validated in Iranian patients with infertility by Omani-Samani et al. (12, 19). A Cronbach's alpha of 70% was obtained for all domains. Exploratory factor analysis revealed five factors for the instrument.

The Female Sexual Function Index (FSFI) consists of 19 questions for assessing the sexual function of women. These questions are organized into six dimensions: sexual desire (two questions), arousal (four questions), lubrication (four questions), orgasm (three questions), sexual satisfaction (three questions), and pain during intercourse (three questions). A six-item Likert scale is used for scoring this index with zero indicating that women did not have sexual intercourse during the last month and five indicating women always having sexual problems. The scores of each domain are multiplied by a certain factor. The minimum and maximum scores for this questionnaire are 6 and 36, respectively, and scores of more than 26 indicated favorable sexual function (14). The Persian version of this questionnaire was validated in Iran by Fakhri et al. (15). The test–retest reliability of FSFI was confirmed by obtaining 0.73-0.86, and this tool was shown to be internally consistent. Confirmatory factor analysis confirmed the validity of all domains of FSFI.

The International Index of Erectile Function (IIEF) is an index for assessing the sexual function of male participants in the past four weeks. This questionnaire consists of five dimensions as follows: erectile function (six questions), orgasmic function (two questions), sexual desire (two questions), sexual satisfaction (three questions), and overall satisfaction of sexual function (two questions). These questions are scored from zero to five, with higher scores indicating better sexual function. The minimum and maximum scores in this questionnaire are 15 and 75, respectively. Scores between 15 and 25 indicated low sexual function, scores between 25 and 50 indicated moderate sexual function, and scores >50 indicate good sexual function (13). The validity and reliability of this questionnaire were assessed and approved by Pakpour et al. (16) in Iran.

### Procedure

All eligible women and men were approached in the infertility clinic of the hospital (Imam Khomeini). After informed consent was obtained from the participants, they were requested to complete three questionnaires. The female participants completed a demographic questionnaire, FSFI, and the Fertility Problem Inventory, while the male participants completed a demographic questionnaire, IIEF, and Fertility Problem Inventory. One of the researchers (SA) was available in case participants had any questions.

## Statistical Analysis

Statistical analysis was performed using the statistical software SPSS 22 (SPSS Inc. Chicago, IL, United States). The normality of continuous variables was examined using the Shapiro-Wilk's *W*-test. Continuous variables were reported as mean ± *SD* whereas categorical data were expressed as numbers (percentage). An independent-samples *t*-test was used to compare the mean of different infertility stress scopes of infertile men and women. In addition, multiple linear regression analysis was applied to determine the parameters most predictive of the infertility stress in men.

A step-wise backward regression algorithm was used to choose variables entering into the final model. All variables that had *p*-values less than 0.1 in a univariate analysis were selected to be examined in this algorithm. In addition, univariate and multivariable binary logistic regression analyses were performed to calculate crude and adjusted odds ratios of the association between the explanatory variables and the sexual function status in infertile women, with the respective 95% CI and *p*-values (likelihood ratio statistic). Variables associated with the outcome (good sexual function/ undesirable sexual function) with *P* < 0.2 in the univariate analysis were included in a multivariate logistic regression model. Backward stepwise logistic regression modeling was then used to obtain a subset of the factors associated with the status of sexual function in infertile women.

## Results

Table 1 shows the demographic characteristics of infertile men and women participating in this study. The mean age of infertile women and men was 29.6 ± 5.5 and 37.7 ± 7.7, respectively. The duration of infertility was 5.6 ± 3.5 and 7.2 ± 3.49 in infertile women and men, respectively. Almost half of the infertile women and men were able to pay for the infertility treatment costs.

**Table 1:** Demographic characteristics of infertile men and women.

**Variables**	**Infertile women** ***n* =150**	**Infertile men** ***n* = 150**
	Mean ± SD	
Age (y)	29.6 ± 5.5	37.7 ± 7.7
Age of spouse (y)	32.3 ± 5.3	31.6 ± 4.8
Length of infertility (y)	5.6 ± 3.5	7.2 ± 3.49
	N(%)	
**Education**		
High school	91(60.7)	90(60)
University degree	59(39.3)	60(40)
**Employment status**		
Employee	66(44)	137(91.3)
Homemaker	84(56)	13(8.7)
**Employment of spouse**		
Employee	150(100)	69(46)
Homemaker	0	78(52)
**Economic status**		
Good	23(15.3)	115(76.7)
Moderate	88(58.7)	35(23.3)
Weak	39(26)	0
**Ability to pay for the infertility treatment costs**		
Yes	89(59.3)	87(58)
No	61(40.7)	63(42)

Table 2 shows the mean sexual function scores in female and male infertile patients. The total score of sexual function in women and men was 22.18 ± 7.86 and 57.7 ± 17.8, respectively.

**Table 2:** Sexual function in infertile women and men.

**Sexual function in women**		**Sexual function in men**	
**Variables**	**Mean ±SD**	**Variables**	**Mean ±SD**
Sexual desire	3.47 ± 1.15	Erectile function	24.2 ± 7.98
Sexual arousal	3.46 ± 1.37	Reach the peak of pleasure	7.74 ± 2.5
Lubrication	3.83 ± 1.43	Sexual desire	7.24 ± 1.9
Orgasm	3.70 ± 1.46	Satisfactory sexual relationship	11.5 ± 3.9
Sexual satisfaction	3.84 ± 1.67	Sexual satisfaction	7.09 ± 2.33
Pain during intercourse	3.69 ± 1.41	Total score sexual function of men	57.7 ± 17.8
Total score sexual function	22.18 ± 7.86		

Table 3 shows the mean infertility stress in infertile women and men. The scores of concerns about a child-free lifestyle and the need for parenthood were significantly higher in infertile women compared to infertile men.

**Table 3:** Comparison of mean scores of different areas of infertility stress in infertile women and men.

**Variables**	**Infertile women** ***n* = 150**	**Infertile men** ***n* = 150**	***P*-value**
	**Mean** **±SD**		
Social concern	38.05 ± 14.04	35.16 ± 7.02	0.41
Sexual concern	28.7 ± 13.1	27.2 ± 6.5	0.08
Communication concerns	35.08 ± 9.6	35.04 ± 5.7	0.66
Concern about child-free lifestyle	31.04 ± 4.9	29.12 ± 4.5	0.001
Need for parenthood	42.6 ± 6.4	36.8 ± 7.6	<0.0001
Total number	175.5 ± 45.7	163.4 ± 25.9	0.06

There was a significant association between the total score of infertility stress with a total score of sexual function in both male and female genders. The total score of sexual function in men was negatively correlated with the total score of infertility stress (*r* = −0.292; *p* < 0.05). In infertile women, the total score of infertility stress has a negative association with the total score of sexual function (*r* = −0.857; *p* < 0.0001) (data are not shown in tables).

Good sexual function was observed in 67 (44.6%) infertile women. The short infertility duration was a significant variable in relation to better sexual function (OR: 0.46; 95%CI: 0.35–0.58; *P* < 0.0001). Other variables had a significant effect on the sexual function of infertile women (Table 4). Results of multivariate logistic regressions for the prediction of good sexual function in infertile women are reported in Table 4. Of all 10 adjusted variables in the multivariate logistic regression model, infertility duration, ability to pay for infertility treatment, and sexual concerns had a significant effect on good sexual function. Infertile women with the highest sexual concern had experienced undesirable sexual function (OR Adjusted: 0.86; 95% CI: 0.79–0.93; *P* < 0.0001).

**Table 4:** Results of univariate and multiple logistic regression models for predicting good sexual function in infertile women.

**Variables**	**Women with good sexual function** ***N* = 67**	**Women with undesirable sexual function *N* = 83**	**Crude OR (95% CI)**	***P***	**Adjusted OR (95% CI)**	***P***
	**Mean** **±SD**					
Age (y)	25.9 ± 4.08	32.6 ± 4.6	0.72(0.656–0.802)	<0.0001		
Age of spouse (y)	40.09 ± 4.02	35.4 ± 4.05	0.67(0.59–0.757)	<0.0001		
Infertility duration (y)	3.09 ± 1.67	7.76 ± 3.29	0.46(0.35–0.58)	<0.0001	0.68 (0.50–0.93)	0.016
**Education; no (%)**				<0.0001		
High school	30(44.8)	61(37.5)	Ref			
University degree	37(55.2)	22(26.5)	3.42(1.72–6.78)			
**Occupation; no (%)**				<0.0001		
Housewife	25(37.3)	59(71.1)	Ref			
Employee	42(62.7)	24(28.9)	4.13(2.08–8.19)			
**Economic status**				<0.0001		
Weak	1(1.5)	38(45.8)	Ref			
Good	66(98.5)	45(54.2)	55.73(7.38–420.7)			
**Ability to pay for infertility treatment costs**				<0.0001		0.020
No	5(7.5)	56(67.5)	Ref			
Yes	62(92.5)	27(32.5)	25.7(9.27–71.34)		11.74 (1.48–93.08)	
**Infertility stress scopes**						
Social concern	24.71 ± 7.59	48.8 ± 7.03	0.790 (0.740–0.848)	<0.0001		
Sexual concerns	16.2 ± 7.41	38.8 ± 6.17	0.792 (0.74–0.84)	<0.0001	0.86(0.79–0.93)	<0.0001
Communication concerns	26.45 ± 5.17	42.06 ± 6.04	0.745 (0.687–0.80)	<0.0001		
Concern about child-free lifestyle	27.2 ± 3.12	34.09 ± 3.92	0.637 (0.55–0.727)	<0.0001	0.85(0.69–1.03)	0.104
Need for parenthood	37.01 ± 4.72	47.17 ± 3.27	0.626 (0.54–0.718)	<0.0001		
The total number of infertility stress	131.6 ± 22.9	211 ± 22.8	0.930 (0.91–0.94)	<0.0001		

Univariate logistic regression indicated that infertile women with higher scores in all scopes of infertility stress were significantly more likely to have undesirable sexual functions (Table 4). Per one-unit increase in score of social concern, sexual concern, communication concern, concern about life without a child and need to be parent scopes, infertile women had a 21, 20.8, 25.5, 36.3, and 37.4% reduction in odds of having good sexual function, respectively (Table 4).

In addition, the multivariate logistic analysis indicated that infertile women with higher scores of sexual concern after adjustment for infertility duration, ability to pay for infertility treatment, and a score of concerns about a child-free lifestyle, were at higher risk of undesirable sexual function (OR = 0.86; 95% CI: 0.79–0.93; *p* < 0.001).

In simple regression analyses, all scopes of infertility stress were correlated negatively with sexual function in infertile male participants. Per one-unit increase in score of social concern, sexual concern, communication concern, concern about child-free lifestyle and need to parenthood, sexual function of infertile men shows reduction as follows: social concern (β = −0.63; *p* = 0.002), sexual concern (β = −0.82; *p* < 0.0001), communication concern (β = −0.95; *p* < 0.0001), concern about child-free lifestyle (β = −1.04; *p* = 0.001), and the total score of infertility stress (β = −0.24; *p* < 0.001) (Table 5).

**Table 5:** Results of univariate and multiple linear regression analyses to determine parameters most predictive of sexual function in infertile men.

**Variables**	**Beta (S.E)**	**95% CI for Beta**	***P***	**Beta (S.E)**	**95% CI for adjusted Beta**	***P***
Age (y)	0.19 (0.18)	(−0.18,0.56)	0.308			
Age of wife (y)	0.20 (0.30)	(−0.39,0.80)	0.496			
Infertility duration (y)	−0.08 (0.42)	(−0.92,0.74)	0.836			
**Education; no (%)**			0.062			
High school	Ref	-		Ref	–	0.091
University degree	5.47 (2.93)			4.79 (2.81)	(−0.76,10.34)	
**Wife's occupation; no (%)**			0.298			
Housewife	Ref	–				
Employee	3.06 (2.94)					
**Infertility stress scopes**						
Social concern	−0.63 (0.202)	(−1.03, −0.23)	0.002			
Sexual concerns	−0.82 (0.212)	(−1.24, −0.40)	<0.0001	−0.46 (0.26)	(−0.98, −0.05)	0.076
Communication concerns	−0.95 (0.24)	(−1.43, −0.46)	<0.0001	−0.61 (0.30)	(−1.21, −0.02)	0.041
Concern about child-free lifestyle	−1.04 (0.31)	(−1.66, −0.43)	0.001			
Need for parenthood	−0.24 (0.19)	(−0.61,0.13)	0.209			
The total score of infertility stress	−0.198 (0.05)	(−0.30, −0.09)	<0.001			

Multiple linear regression analysis demonstrated that communication concern was significantly correlated with lower levels of sexual function in men after controlling the effects of other predictors (β = −0.61, 95% CI: (−1.21, −0.02) (Table 5).

## Discussion

This study was designed to investigate the relationship between infertility stress and sexual function in infertile women and men. The results of this study showed that most infertile women had a low sexual function score, while most infertile men had good sexual function. There is evidence that infertility can negatively affect sexual function in women and men, and 43–90% of infertile women and 48–58% of infertile men have been reported to suffer from sexual dysfunction (17). Tao et al. (18) also suggested that infertility and its treatment could negatively affect the sexual function dimensions such as sexual self-esteem and sexual relationship. Our results are in line with those of Starc et al. (17) and Tao et al. (18). In a systematic review, Omani-Samani et al. (12, 19) found that 64% of Iranian infertile women had sexual dysfunction. In our study 83 (55.3%) women had an undesirable sexual function, which is almost in line with the study of Omani-Samani et al. (12, 19).

The results of the present study showed that the scores of concerns about a child-free lifestyle and the need for parenthood were significantly higher in infertile women compared to infertile men. In total, 67 (44.6%) of the infertile women in the present study had normal sexual function. Women with shorter infertility duration had better sexual function. In addition, women who could pay for infertility treatment were more likely to have better sexual function. In their study on 300 women with primary infertility, Patel et al. (5) found that almost 80% of infertile women had infertility stress and that duration of infertility and infertility type were among the predictors of stress among these women.

According to the results of the present study, infertile women with higher sexual concerns had an undesirable sexual function. Also, a significant association was found between communication concerns and sexual function scores in infertile men. Factors such as infertility treatment and using assisted reproductive technology, having timed intercourse, and psychological factors may impair sexual function in infertile women (20). Bai et al. found that infertility treatment costs and social concerns were associated with depression in men, while ethnicity and sexual concerns were significantly correlated with depression symptoms in women (21). These results are in line with our findings, except for the fact that infertility treatment costs were related to sexual function in infertile women and not in men. In the present study, infertile women and men were from different families, and no couples participated in this study. Most of the women in this study did not have a good economic status, while most infertile men (76.7%) enjoyed a good economic position.

A study by Salomao et al. (22) showed that infertile women do not have higher sexual dysfunction in comparison to the women without infertility, but women with more anxiety or depression showed more sexual dysfunction. It is not known, however, whether women experience sexual dysfunction due to infertility or depression or if stress following infertility can cause sexual dysfunction. Of course, it is hardly disputable that infertility is a complex problem and has many negative effects on various organs (23).

Sexuality is a complex phenomenon, and there is a need for more studies to prove the causal relationship between infertility, stress, and sexual dysfunction in infertile women and men.

### Strengths and Limitations of the Study

In this study, we assessed the relationship between infertility stress and sexual function among 300 infertile women and men. One of the limitations of this study is that the participants were not recruited randomly. Second, we did not have a control group. Finally, the questions in the questionnaires required the participants to answer based on their memory, and thus the results may have been affected by recall bias.

## Conclusion

This study showed that infertile women suffer from higher infertility stress than do infertile men. Women with higher infertility duration and inability to pay for the infertility treatment costs had impaired sexual function. Infertile women had more sexual concerns, while infertile men had more communication concerns. Policymakers need to consider strategies such as counseling for infertile men and women to help them cope with their problems, especially their psychological problems.

## Data Availability Statement

The raw data supporting the conclusions of this article will be made available by the authors, without undue reservation.

## Ethics Statement

The studies involving human participants were reviewed and approved by Ahvaz Jundishapur University of Medical Sciences. The patients/participants provided their written informed consent to participate in this study.

## Author Contributions

SA, PA, RN, and MT were contributed to the conception of this study. SA collected the data. SA and EM were contributed to data analyzing. SA, EM, and PA were contributed in data interpretation. PA was responsible for writing the paper. All authors seen and approved the content of the manuscript.

## Funding

This study was funded by Ahvaz Jundishapur University of Medical Sciences, Ahvaz, Iran. The funder did not play any role in conception, data collection, data interpretation, and writing this manuscript.

## Conflict of Interest

The authors declare that the research was conducted in the absence of any commercial or financial relationships that could be construed as a potential conflict of interest.

## Publisher's Note

All claims expressed in this article are solely those of the authors and do not necessarily represent those of their affiliated organizations, or those of the publisher, the editors and the reviewers. Any product that may be evaluated in this article, or claim that may be made by its manufacturer, is not guaranteed or endorsed by the publisher.
